# An Enhanced Self-Care Protocol for People Affected by Moderate to Severe Lymphedema

**DOI:** 10.3390/mps2030077

**Published:** 2019-09-04

**Authors:** Janet Douglass, Hayley E. Mableson, Sarah Martindale, Louise A. Kelly-Hope

**Affiliations:** Centre for Neglected Tropical Diseases, Department of Tropical Disease Biology, Liverpool School of Tropical Medicine, Liverpool L3 5QA, UK

**Keywords:** lymphedema, lymphatic-filariasis, podoconiosis, self-care, lower-limb, exercise, massage

## Abstract

Lymphedema is a chronic skin disease that has many causes and leads to significant disfigurement and disability worldwide. Recommendations for lymphedema self-care vary by setting and the World Health Organization guidelines for people affected by lymphatic filariasis- and podoconiosis-related lymphedema are centered around a basic daily hygiene regimen. Research on cancer-related lymphedema in developed country settings suggests that deep-breathing exercises and self-massage can improve lymphedema status, but these exercises are not routinely taught to people affected by lymphedema in developing country settings. To determine if the activities proven in cancer-related lymphedema can improve outcomes for people affected by lymphatic filariasis- or podoconiosis-related lymphedema, an enhanced self-care protocol for lower limb lymphedema was developed and trialed in Nilphamari District in Bangladesh and Simada Woreda in Ethiopia. Enhanced self-care activities were chosen on the basis that they would not add financial burden to patients or their families and included recommendations to perform deep-breathing exercises and self-massage, drink clean water, and eat fresh fruits and vegetables. The enhanced-care protocol was developed in collaboration with implementing partners in both countries and may be applicable in other populations affected by lower-limb lymphedema. Trial methods and results will be submitted for peer reviewed publication. Current recommendations for lymphedema self-care may be less effective for people with more advanced disease and new or cross-cutting methods are needed to improve outcomes for these populations.

## 1. Introduction

Lymphedema is a chronic swelling of the skin and underlying tissue and a major cause of disability globally. It can occur from multiple etiologies including lymphatic filariasis (LF), a vector-borne parasite endemic to many tropical countries [1] and podoconiosis is lymphedema caused by long-term bare-foot exposure to irritant soils [2]. It is estimated that between these two diseases, more than 20 million people live with the daily burden of lymphedema, predominately affecting the lower limbs [2,3]. This burden extends to families and communities and is a major contributor to poverty in many developing countries [4]. The Global Programme to Eliminate Lymphatic Filariasis (GPELF) as a public health problem is a World Health Organization (WHO) mandated agreement between endemic countries. Member states are required to deliver preventive chemotherapy via mass drug administration campaigns to all people at risk of infection, and to provide a basic package of care for people with existing morbidity (lymphedema and hydrocele) [5]. This basic package is referred to as morbidity management and disability prevention (MMDP) and should be integrated into the national health system, with lymphedema services delivered through community health centers [6]. Whilst there is no mandated elimination program for podoconiosis, it is none-the-less a public health problem in endemic countries and the WHO recommends that podoconiosis should be managed within LF MMDP programs [2]. 

The lymphatic system works alongside the venous system to return fluid from the periphery to the heart. The majority of lymph is formed at the initial lymph plexus, a network of fine vessels under the skin where it plays an important role in barrier defenses. The formed lymph passes into collecting vessels which actively pump the lymph towards lymph nodes where it is processed by immune cells. Lymphedema forms when lymphatic clearance fails and can occur in any body part. Untreated lymphedema progresses through identifiable stages and there are several staging systems in common use [7]. In the ‘mild’ stages of lymphedema distal swelling which may have initially reversed on elevation or overnight can become more persistent and progress proximally. In the middle or ‘moderate’ stages there is an overgrowth of fibrous tissue and fatty deposits which can increase tissue stiffness. In the advanced or ‘severe’ stages the skin becomes thickened and forms into deep folds.

In LF, mosquitoes transmit microfilariae between human hosts where they are picked up by the initial lymph plexus under the skin. Mature worms form ‘nests’ in the lymph vessels close to lymph nodes and reproduce. A cascade of host responses to worm products and their symbiotic bacteria dilate the lymph vessels and inhibit lymphatic pumping, eventually leading to lymphatic failure [8,9]. Although the disturbance is usually in vessels located near the groin, the swelling appears first at the ankles and feet. Podoconiosis also results in lymphatic failure when irritant minerals in soils of volcanic origin [10,11] are absorbed into the lymph plexus underlying the skin of the foot. Over time the chronic inflammatory reactions that occur damage the vessels and lymphatic failure follows. As in LF, the swelling appears first in the feet and ankles but due to the site exposure of the irritant, skin changes on the feet can occur much earlier in podoconiosis than in LF-related lymphedema. 

In both diseases a major portion of the burden to patients and their communities is due to the frequency, intensity, and duration of secondary bacterial and fungal infections also known as ‘acute attacks’ [12,13,14]. These debilitating and painful episodes reduce the person’s capacity to contribute to family resources and may affect other family members who are kept from work or school to attend the sick patient [15,16]. Wounds and ulcers (entry lesions) and skin breakdown between the toes (interdigital lesions), are a major source of these infections. A daily home-based hygiene protocol is effective in reducing acute attacks and central to lymphedema management in resource poor settings [17]. This involves daily washing and drying of affected body parts, passive range of motion exercises and calf pump exercises, and elevation of the affected limbs overnight and whenever possible during the day [6]. 

Two systematic reviews on home-based self-care for lymphedema [17,18] demonstrated that a hygiene-centered self-care protocol can reduce the frequency and duration of acute episodes at all stages of disease. In mild stages, swelling may also reverse, but people affected by moderate or severe lymphedema are less likely to experience any reversal of limb size or lymphedema stage. One review compared self-care for people affected by lymphedema from LF with self-care for people affected by lymphedema after cancer therapy [18]. Simple measures such as deep breathing, self-massage, and progressive exercises were shown to benefit women with breast cancer-related lymphedema of the arm [19,20]. These activities aim to stimulate lymph flow from the affected area and are emphasized equally to recommendations for meticulous skin care. In contrast, MMDP guidelines for LF- and podoconiosis-related lymphedema offer minimal stimulation to the lymph vessels themselves [6,21]. 

To determine if the activities proven in cancer-related lymphedema can improve outcomes for people affected by LF- or podoconiosis-related lymphedema, an enhanced self-care protocol for lower limb lymphedema was developed and trialed in Nilphamari District in Bangladesh and Simada Woreda (district) in Ethiopia. Enhanced self-care activities were chosen on the basis of previous evidence [9,10] and expert opinion [22] and with the criterion that they not add any financial burden to the local health services, patients, or their families. The additional activities were designed to stimulate lymph flow, support immune function, and improve skin integrity, and included deep breathing exercises, leg exercises, self-lymphatic-massage, and recommendations to drink clean water and eat fresh fruits and vegetables. 

## 2. Enhanced-Care Protocol Development and Study Approval

The enhanced-care study was developed in accordance with the Standard Protocol Items Recommendations for Interventional Trials (SPIRIT) checklist [23]. Members of the Centre for Neglected Tropical Diseases, Liverpool School of Tropical Medicine, UK, designed the study in consultation with Bangladesh and Ethiopia collaborators. The self-care protocols were developed by the principal investigator (JD) who has expertise in lymphedema management. In Bangladesh, staff from the Ministry of Health and Family Welfare (MOHFW) and the Center for Injury Prevention and Research, Bangladesh (CIPRB), and in Ethiopia, staff from the Federal Ministry of Health (FMOH) and the National Podoconiosis Action Network (NaPAN), provided country specific advice on delivery of the protocols and study execution. The final protocol was approved by the Liverpool School of Tropical Medicine Research Ethics Committee (approval number 012-18), the Bangladesh Human Medical Research Committee, and the Amhara Public Health Institute Research Ethics Committee. The study was registered on the ISRCTN Registry, trial number 16764792 and in accordance with the Declaration of Helsinki [24] and all participants gave their informed consent before inclusion in the study. The study sponsor and funders had no role in study design or protocol development and will have no input into publication of study outcomes. 

## 3. Study Design and Participants

### 3.1. Study Design

**Figure 1:**
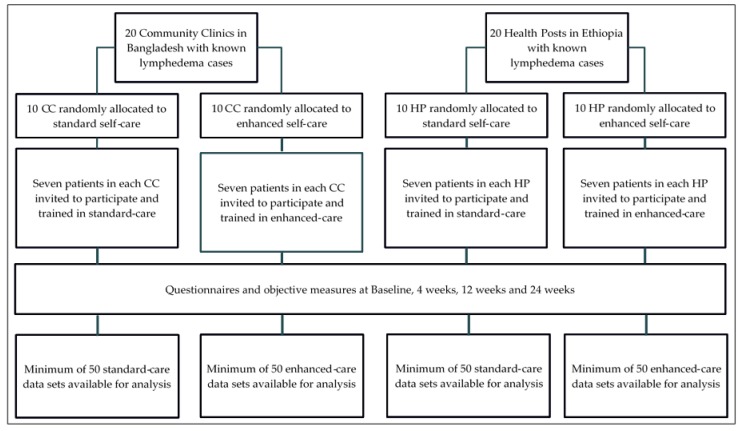
Flow chart showing the study design. CC = community clinic, HP = health post.

Cluster randomization was used to select 20 community clinics (CC) in Nilphamari District and 20 health posts (HP) in Simada Woreda and to allocate each to either the intervention or control group (Figure 1). Adults aged 18 or over were eligible to participate if they had lymphedema in at least one leg at Stage 3 or higher according to the Dreyer stage criterion which describe the identifying features of seven stages [25].

### 3.2. Outcomes

Participants were interviewed at baseline and on exit to elicit demographic information, medical and lymphedema history, and lymphedema knowledge, attitudes, and practice (KAP). Objective measures and quality of life (QOL) questionnaires were collected at baseline and repeated at four weeks, 12 weeks, and 24 weeks. Primary outcome measures included the change in lymphedema stage, midcalf circumference, midcalf tissue compressibility (an indication of the degree of fibrotic induration), number of entry lesions and interdigital lesions, and frequency and duration of acute attacks. Secondary outcome measures were perceived function, lymphedema KAP, QOL, and adherence to the self-care protocols. All questionnaire responses and biometric measurements were recorded via the Open Data Kit Collect (ODK Collect) application [26] loaded to an electronic tablet (Samsung Galaxy Tab A 10.1). 

### 3.3. Participants at Baseline

In Bangladesh baseline data were collected on 146 patients who were mostly female (n = 107, 73.3 %) with a mean age of 54 years (SD 11.3, range 27–88). Lymphoedema was found in 177 legs (60.6% of all legs) and the median stage was Stage 3 (n = 103 legs (58.2% of affected legs)) and there were 32 legs at Stage 6 (18.1% of affected legs). No-one was affected by leg lymphoedema at Stage 7. Thirty-nine percent of participants had unilateral right leg lymphoedema, 40.4% had unilateral left leg lymphoedema, and 20.6% had bilateral leg lymphoedema. Of the 140 participants who said they had ever had an acute attack, 130 had suffered at least one attack in the previous 6 months. The mean frequency was 4.76 episodes (SD 4.75, median 3 attacks, range 0–24), and the mean duration of each attack was 4.66 days (SD 3.18, median 4 days, range 0–20). 

In Ethiopia baseline data were collected on 129 patients who were relatively evenly distributed by sex (males; n = 70, 54.3%) with a mean age of 58 years (SD 11.467, range 25–84). Lymphoedema was found in 227 legs (88% of all legs) and the median stage was Stage 6 (n = 149 legs (65.6% of affected legs)) and there were 51 legs at Stage 3 (22.5% of affected legs). One person was affected by leg lymphoedema at Stage 7. Unilateral lymphedema was present on the right leg of 16.3% of participants, 7.8% had unilateral left leg lymphoedema, and 75.9% had bilateral leg lymphoedema. Of the 103 participants who said they ever had an acute attack 92 people had suffered at least one attack in the previous 6 months. The mean frequency was 3.31 episodes (SD 3.96, median 3 attacks, range 0–34), and the mean duration of each attack was 4.64 days (SD 5.133, median 3 days, range 0–29).

## 4. Lymphedema Self-Care Protocols

### 4.1. Standard-Care 

The self-care protocol for the control group followed the MMDP recommendations for community-based home-care in LF-related lymphedema [6]. This included education in the relevant causes of lymphedema, hygiene and skin care practices for the feet and legs, seated exercises (range of motion exercises at the ankle), standing exercises (raising the body weight up and down on the toes), daily and overnight elevation of the affected limb(s), and management of acute attacks. All exercises were given in sets of five movements with each leg and participants were advised to do them at least twice per day, more often if possible. A brief description of each activity in the standard-care protocol is given in Table 1 and a full description of each activity follows.

#### 4.1.1. Hygiene and Skin Care 

Thorough and frequent washing and drying of affected body parts is central to the success of lymphedema self-care, and this was described to the participants as special washing for their lymphedema, not usual washing, and not because they were dirty or unclean. Washing was performed by placing the feet in a collecting bowl and pouring a little clean, room temperature water, over the legs. Plain soap (pH < 7) was used to create a soapy lather in the hands and this was applied to the leg below the knee in a gentle downward motion to loosen surface dirt and bacteria. The soap was rinsed off with clean water. The wash cloth was then dampened and lathered with the soap and used to clean between skin folds and toes. The cloth was folded to create a double edge which could be inserted into a skin fold or interdigital space and used in a sawing motion, being careful of any painful interdigital lesions. The cloth was refolded to provide a clean part for each new skin fold or toe space. Both the cloth and toe spaces were inspected during the process to make sure all dirt was removed and the cloth was rinsed and re-lathered until no further dirt remained. The necessity to remove all dirt, bacteria, and fungus from skin folds and toes spaces was emphasized in relation to the onset of acute episodes. After thorough rinsing to make sure no soap remained in the skin folds and toe spaces, careful drying was demonstrated using a similar folded towel technique. The need to thoroughly dry between the toes and skin folds to reduce fungal infections was emphasized. Both legs and feet were thoroughly washed and dried beginning with the most affected leg. On days when the toe nails were to be clipped it was recommended that that the toes be left in the water for a few minutes to soften the nails first. 

After washing and drying, the toe spaces were inspected for any interdigital lesions and wherever these were present antifungal cream was applied deep into the interdigital space, being careful once again to avoid causing any pain. If there were entry lesions on the foot or leg these were also treated with either antibacterial cream (Bangladesh) or petroleum jelly (Ethiopia). In Bangladesh entry lesions could also be covered with a gauze bandage (supplied) and in Ethiopia the petroleum jelly was used to prevent flies landing on any open wounds. 

Participants were advised to wash and dry both legs and attend to entry lesions morning and night, and at night to also wash any footwear used that day.

#### 4.1.2. Seated Exercises

These were performed sitting on a chair, low stool, or bench and varied slightly between countries. In both countries, participants were shown how to mobilize the ankle passively and actively. Passive movements were made by placing one ankle on the opposite knee and clasping the toes with the opposite hand. The hand was then used to describe circles with the foot in each direction moving the ankle through its comfortable range of motion (ROM). The circles were performed at least five times in each direction and with both legs, even if one leg was unaffected. Active ankle exercises were performed with the leg extended and the heel lifted off the floor, supporting the leg with the hands clasped behind the thigh if needed. The ankle was flexed up and down and also moved from side to side. Sets of five repetitions for each movement were recommended and were to be done with both legs. 

In Bangladesh participants were also shown two more seated exercises which are routinely taught during MMDP campaigns. The first involved straightening one leg and reaching forward with the hand on the same side to pull the ankle back, bending the knee to bring the heel toward the outside of the thigh and buttock. The position was not held but returned to the starting position and repeated. This movement stretches the large muscles on the front of the thigh. The second exercise involved clenching and extending the toes to exercise the calf muscles. To achieve this a long cloth was laid on the floor extending away from the feet. With bent knees and soles flat to the floor, the toes of both feet were used to grip the near edge of the cloth and draw it back towards the body in small sections. The toes were extended to grip the next section of cloth and the action was continued until the far edge was drawn in. 

All exercises were given in sets of five movements with each leg and participants were advised to do them at least once per day, more often if possible. 

#### 4.1.3. Standing Exercises

Participants were shown how to rise up and down on their toes to activate the calf muscles. They were encouraged to do this slowly to feel the muscles working. Participants who found it difficult to balance were shown how to steady themselves by placing a hand on a wall or chair back, or using a staff to steady themselves. Sets of five toe raises were recommended, at least once per day, more often if possible. 

#### 4.1.4. Elevation

Locally appropriate suggestions were given on ways to elevate the end of the bed or sleeping mat. This included suggestions such as placing a small block of wood under the legs at the end of a bed, using flattened, partially filled sacks under the end of a sleeping mat, or placing pillows under the legs. Day time elevation was also encouraged whenever possible by placing the limb on a stool, cushion or other supportive surface with the foot higher than the hip.

#### 4.1.5. Managing Acute Attacks

Participants were informed of the symptoms of acute attacks and advised to stop performing the exercise and massage components of their self-care protocols, whilst maintaining the hygiene elements. It was recommended that limbs that felt hot be cooled in buckets of cool water or with cool cloths. Topical and oral antibiotics, anti-inflammatories, and pain medications were recommended according to need and availability. Mild and moderate symptoms were to be managed at home and help sought at the local health service for more severe episodes.

#### 4.1.6. Support services

Participants were advised on what services could be accessed locally and encouraged to form self-help groups or seek out and talk with other people affected by lymphedema.

### 4.2. Enhanced-Care 

The intervention group were given the same standard-care training as described above, with the addition of the enhanced-care activities to support lymph flow. These included deep abdominal breathing, leg exercises performed whilst lying supine, 45 minutes of walking, massage of the legs with or without oil, and mobilization of hardened lymphedema skin and tissue. Recommendations were also given regarding drinking water and eating fresh fruits and vegetables which aimed to support immune system functions. A brief description of each of the additional activities is given in Table 2 and a full description of the additional activities follows.

#### 4.2.1. Deep Breathing Exercises 

Referred to clinically as the thoraco-abdominal pump, diaphragmatic breathing creates a pressure variation between the thoracic and abdominal cavities which favors fluids such as blood and lymph moving towards the heart. To learn the exercise, participants were asked to place one hand on their abdomen and one hand on the upper chest. They were instructed to take a deep breath and imagine that the breath was being pulled down into their belly without allowing the chest to rise. Attention was drawn to their hands, so they could feel the belly hand being pushed out without the chest hand moving very much. This is a difficult exercise to master initially, but with only a little practice becomes automatic. Five breaths on five occasions during the day were recommended. Deep breathing can be performed standing, sitting, or lying down.

#### 4.2.2. Lying Down Exercises

Performed in conjunction with deep breathing and lying supine on a bench, sleeping mat or the floor with the knees bent and feet flat to the floor, a pillow can support the head if necessary. Two exercises were performed in this position, both legs, five repetitions for each exercise on each leg. In the first exercise the hands were used to pull one knee firmly towards the chest, compressing the abdomen during forced exhalation. The foot was returned to the floor during the inhalation. In the second exercise one leg was extended at the knee and the foot lifted up and down without touching the floor. The hands were clasped behind the thigh to support the leg if necessary.

#### 4.2.3. Skin and Tissue Mobilization 

Overgrowth of subcutaneous fibrous tissue is difficult to reverse but can be gradually softened with gentle and frequent tissue mobilization. This was demonstrated by rolling the large muscles around the bones of the thigh and calf in a transverse direction. Comparison was drawn between and normal skin area such as an unaffected thigh where the skin and muscle move freely, and lymphedema tissue which is stiff and immobile. Over the less flexible areas a flat hand was used to move the skin back and forth in relation to the underlying tissue. Movements were done firmly but gentle enough not to cause pain or undue discomfort. These gentle mechanical actions create shearing forces into the connective tissue which can soften the dense fibrous deposits. Skin mobilization can be performed before or during lymphatic massage and can be repeated throughout the day whenever convenient.

#### 4.2.4. Lymphatic Massage 

An effleurage technique (stroking) was used in a hand-over-hand motion in the direction of lymph flow (distal to proximal). The lymph vessels are close to the surface of the skin therefore the pressure is light with the emphasis on a flat hand to sweep the fluid along, rather than pressing downward into the muscles. Deep breathing was done first to ‘pull’ fluid up out of the abdomen into the chest and then the flat effleurage strokes were used along the skin of the inner thigh (over clothing if necessary) directing fluid from above the knee towards the lymph nodes in the groin. Each stroke was done several times. Next the strokes were applied along the inside of the knee with the same upward hand-over-hand movement, and then the shin and calf were stroked up towards the knee. The foot and toes were stroked up onto the ankle. One leg was treated at a time beginning with the least affected leg. At least one massage per day was recommended, ideally after washing and drying the legs. The massage strokes below the knee were demonstrated using a small amount of edible vegetable oil but the option to perform all movements without oil was also given.

#### 4.2.5. Walking, Drinking Water, and Eating Fresh Fruits and Vegetables

People affected by severe lymphoedema often develop sedentary lifestyles and restrict their water intake which may contribute to lymphoedema progression. Current recommendations to achieve between 3000 and 10,000 steps each day to maintain health were considered in determining the minimum target for daily walking [27]. To meet this goal, participants were advised to walk for at least 45 minutes per day (not necessarily all at once) to increase or maintain their mobility, and to drink at least five glasses of clean water every day. 

Good nutrition is essential to wound healing and it was recommended that participants eat at least one serve of fresh fruit or vegetables on at least 4 days every week if possible [22].

## 5. Participant Training

Lymphedema management in resource poor settings usually relies on community-based home-care. Typically, patients will be identified by local health authorities or non-governmental agencies and shown how to perform the hygiene routine and other self-care recommendations including advice on management of acute attacks. How this information is distributed, and what local resources are available to patients, may vary from country to country and region to region. In this study, training in the standard-care activities followed usual implementation of MMDP in each country. Education in locally relevant risk factors was given, and risk reduction behaviors were encouraged. This included advice such as the use of bed nets in LF endemic areas, and wearing shoes and covering dirt floors in podoconiosis endemic areas. In centers allocated to the enhanced-care intervention, trainers included the additional activities within the self-care training session. 

Each activity was carefully explained and demonstrated by the trainer(s), including information about the purpose of each component and suggestions for integrating self-care activates into normal daily routines. A time frame for performing daily self-care was not specified to the participants, rather the formation of habits was encouraged. Participants in both groups were instructed not to cause pain with any of the limb care activities, but to do things as gently as needed to remain comfortable. At the end of the study participants who had been allocated to the standard-care group were offered training in the additional enhanced-care activities.

### Material and Resources

Participants were provided with materials in keeping with what would usually be supplied to patients affected by lymphedema in each country, therefore there were some small variations between countries in what was supplied. In Bangladesh the Filariasis Elimination Program offers a kit box of materials which includes wash cloths, towels, medicated creams and gauze bandages. This was modified slightly to add nail clippers (both groups) and coconut oil for the massage (enhanced-care group only). The kit box was supplied at the beginning of the study and again at 12 weeks In Ethiopia patients were supplied with a wash bowl, wash cloths, towels, medicated creams, nail clippers and vegetable oil (enhanced-care group only) at the beginning of study. The soaps and medicated creams were replenished at every follow-up. A list of materials supplied in each country is given in Table 3. A color brochure with pictures of the allocated self-care activities was provided to participants in local language and examples for each group are provided in Supplementary materials Figure S1: Standard-care brochure Bangladesh, and Figure S2: Enhanced-care brochure Ethiopia. Participants were advised on what services could be accessed locally during acute attacks and when materials for the study would be replenished. 

## 6. Expected Results

It is expected that both protocols will achieve reduction in the frequency and duration of acute attacks and improve quality of life among people affected by lymphedema. It is anticipated that data analysis will reveal increased improvement in lymphoedema status in the enhanced-care group compared to their peers who were performing the recommended MMDP protocol (standard-care). The enhanced-care protocol could be easily transferred to other LF and podoconiosis endemic settings or other populations affected by lower limb lymphedema. 

## 7. Future Directions

Effective self-care training for people living with lymphedema must be sustainable and take into account available resources, or in most places, the lack of personal means or adequate support services. This poses challenges for LF- and podoconiosis-endemic countries in implementing MMDP programs and has led to a minimalist approach in lymphedema management. Current MMDP recommendations may be less effective for people with more advanced disease and new or cross-cutting methods are needed to improve outcomes for these populations, without adding further burden on the family or community. This enhanced-care protocol was designed with these restraints in mind. Statistical analysis on results of the trial will be submitted for peer reviewed publication. 

## Figures and Tables

**Table 1:** Standard-care activities.

Activity	Description	Frequency
Leg washing and drying	Thoroughly wash and dry both legs including skin folds and interdigital spaces	≥ twice per day
Attending to entry lesions	Careful inspection of legs, feet and toe spaces to identify broken or damaged skin	≥ twice per day
Applying medicated cream	Apply antifungal cream to interdigital lesions Apply antibiotic cream (Bangladesh) or petroleum jelly (Ethiopia) to entry lesions	≥ twice per day
Trimming nails	Trim broken or long toenails	As needed
Standing exercises	Slowly rise up and down on the toes	≥ once per day
Seated exercises	Passive ROM exercises for the ankle Active ROM exercises for the ankle Sets of 5 in each direction with each leg	≥ once per day
Daytime elevation	Place the affected leg(s) on a stool or cushion so the foot is higher than the hip	Whenever sitting
Night-time elevation	Raise the end of the bed or sleeping mat so the feet are higher than the head	Every night
Managing acute attacks	Seek symptomatic relief such as cooling a hot limb and taking pain medication Rest and cease the exercises until the attack has resolved, continue all hygiene practices Use medicated cream and take oral medication (antibiotic or anti-inflammatory) if required	As needed
Accessing support services	Attend the health facility for help or advice Attend lymphedema support groups	As needed

ROM = range of motion

**Table 2:** Enhanced care activities (given in addition to all activities in Table 1).

Activity	Description	Frequency
Deep breathing exercises	Inhale deep into the abdomen using the diaphragm so that the belly pushes out without allowing the chest to rise	5 breaths ≥5 times per day
Lying down exercises	Knees bent and using one leg at a time: 1: Draw the knee firmly into the chest during the exhale, returning the foot to the floor during the inhale 2: Lift the lower leg up and down without touching the floor	Repeat each movement 5 times with each leg ≥ once per day
Mobilizing the skin and tissue	Gently move and roll any skin or underlying tissue that feels hard	≥ once per day
Lymphatic massage	Massage both legs beginning with the least affected leg Use a flat hand-over-hand stroking technique in a distal to proximal direction Edible vegetable oil can be used as a massage medium (optional)	≥ once per day
Walking	Walk as much as possible, ideally for ≥45 minutes per day	Daily
Drinking clean water	Drink ≥5 glasses of clean water	Daily
Eating fresh fruits and vegetables	Eat at least once serve of fresh fruit or vegetables	≥4 days per week

**Table 3:** Materials supplied to participants to perform lymphedema self-care. The supplies provided are in keeping with government policy in each country.

Activity	Bangladesh	Ethiopia
Leg washing and drying	Soap* 2 wash cloths 2 drying cloths	Large wash bowl ^1^ Soap* 1 wash cloth ^1^ 1 drying cloth ^1^
Attending to entry lesions	Antifungal cream Antibiotic cream Gauze bandage	Whitfield^®^ cream ^2^ Vaseline^®^
Trimming nails	Nail clippers	Nail clippers ^1^
Lymphatic massage	250 ml coconut oil (enhanced-care group only)	1 litre vegetable oil ^1^ (enhanced-care group only)

* Soaps were less than pH 7 and commercially available, ^1^ supplied only at baseline, ^2^ antifungal cream.

## Supplementary Materials

The following are available online at https://www.mdpi.com/2409-9279/2/3/77/s1, Figure S1: Standard-care brochure Bangladesh, and Figure S2: Enhanced-care brochure Ethiopia

## References

[1] World Health Oganization (2010). Progress Report 2000–2009 and strategic plan 2010–2020 of the global programme to eliminate lymphatic filariasis: Halfway towards eliminating lymphatic filariasis. WHO Library Catalogue.

[2] Deribe K., Cano J., Trueba M.L., Newport M.J., Davey G. (2018). Global epidemiology of podoconiosis: A systematic review. PLoS Negl. Trop. Dis..

[3] Ramaiah K.D., Ottesen E.A. (2014). Progress and impact of 13 years of the global programme to eliminate lymphatic filariasis on reducing the burden of filarial disease. PLoS Negl. Trop. Dis..

[4] Mackenzie C.D., Lazarus W.M., Mwakitalu M.E., Mwingira U., Malecela M.N. (2009). Lymphatic filariasis: Patients and the global elimination programme. Ann. Trop. Med. Parasitol..

[5] Rio D.F., World Health Organization (1999). Global Programme to Eliminate Lymphatic Filariasis; Annual Report of Lymphatic Filariasis 2000.

[6] WHO (2013). Lymphatic Filariasis: Managing Morbidity and Preventing Disability: An Aide-Mémoire for National Programme Managers.

[7] Douglass J., Kelly-Hope L. (2019). Comparison of staging systems to assess lymphedema caused by cancer therapies, lymphatic filariasis, and podoconiosis. Lymphat. Res. Biol..

[8] Nutman T.B. (2013). Insights into the pathogenesis of disease in human lymphatic filariasis. Lymphat. Res. Biol..

[9] Dreyer G., Addiss D., Roberts J., Noroes J. (2002). Progression of lymphatic vessel dilatation in the presence of living adult *Wuchereria bancrofti*. Trans. R. Soc. Trop. Med. Hyg..

[10] Davey G. (2010). Podoconiosis, non-filarial elephantiasis, and lymphology. Lymphology.

[11] Davey G., Tekola F., Newport M.J. (2007). Podoconiosis: Non-infectious geochemical elephantiasis. Trans. R. Soc. Trop. Med. Hyg..

[12] Jullien P., Somé J.D., Brantus P., Bougma R.W., Bamba I., Kyelem D. (2011). Efficacy of home-based lymphoedema management in reducing acute attacks in subjects with lymphatic filariasis in burkina faso. Acta Trop..

[13] Dreyer G., Medeiros Z., Netto M.J., Leal N.C., de Castro L.G., Piessens W.F. (1999). Acute attacks in the extremities of persons living in an area endemic for bancroftian filariasis: Differentiation of two syndromes. Trans. R. Soc. Trop. Med. Hyg..

[14] Negussie H., Molla M., Ngari M., Berkley J.A., Kivaya E., Njuguna P., Fegan G., Tamiru A., Kelemework A., Lang T. (2018). Lymphoedema management to prevent acute dermatolymphangioadenitis in podoconiosis in northern ethiopia (golbet): A pragmatic randomised controlled trial. Lancet Glob. Health.

[15] Martindale S., Mackenzie C., Mkwanda S., Smith E., Stanton M., Molyneux D., Kelly-Hope L. (2017). “Unseen” caregivers: The disproportionate gender balance and role of females in the home- based care of lymphatic filariasis patients in Malawi. Front. Women's Health.

[16] Cassidy T., Worrell C.M., Little K., Prakash A., Patra I., Rout J., Fox L.M. (2016). Experiences of a community-based lymphedema management program for lymphatic filariasis in odisha state, India: An analysis of focus group discussions with patients, families, community members and program volunteers. PLoS Negl. Trop. Dis..

[17] Stocks M.E., Freeman M.C., Addiss D.G. (2015). The effect of hygiene-based lymphedema management in lymphatic filariasis-endemic areas: A systematic review and meta-analysis. PLoS Negl. Trop. Dis..

[18] Douglass J., Graves P., Gordon S. (2016). Self-care for management of secondary lymphedema: A systematic review. PLoS Negl. Trop. Dis..

[19] Moseley A.L., Piller N.B., Carati C.J. (2005). The effect of gentle arm exercise and deep breathing on secondary arm lymphedema. Lymphology.

[20] Barclay J., Vestey J., Lambert A., Balmer C. (2006). Reducing the symptoms of lymphoedema: Is there a role for aromatherapy?. Eur. J. Oncol. Nurs..

[21] Sikorski C., Ashine M., Zeleke Z., Davey G. (2010). Effectiveness of a simple lymphoedema treatment regimen in podoconiosis management in Southern Ethiopia: One year follow-up. PLoS Negl. Trop. Dis..

[22] Lehman L.F., Geyer M.J., Bolton L. Ten Steps. A Guide for Health Promotion and Empowerment of People Affected by Neglected Tropical Diseases. https://leprosy.org/ten-steps/.

[23] Chan A.-W., Tetzlaff J.M., Gøtzsche P.C., Altman D.G., Mann H., Berlin J.A., Dickersin K., Hróbjartsson A., Schulz K.F., Parulekar W.R. (2013). Spirit 2013 explanation and elaboration: Guidance for protocols of clinical trials. BMJ Br. Med. J..

[24] World Medical Association (2013). World medical association declaration of Helsinki: Ethical principles for medical research involving human subjects. JAMA.

[25] Dreyer G., Addiss D., Dreyer P., Noroes J. (2002). Basic Lymphoedema Management, Treatment and Prevention Problems Associated with Lymphatic Filariasis.

[26] Hartung C., Lerer A., Anokwa Y., Tseng C., Brunette W., Borriello G. Open data kit: Tools to build information services for developing regions. Proceedings of the 4th ACM/IEEE International Conference on Information and Communication Technologies and Development.

[27] Choi B.C., Pak A.W., Choi J.C. (2007). Daily step goal of 10,000 steps: A literature review. Clin. Investig. Med..

