# Supplementary material for: An Enhanced Self-Care Protocol for People Affected by Moderate to Severe Lymphedema
Source: Methods Protoc. 2019 Sep 4;2(3):77. doi: 10.3390/mps2030077 (PMC6789820; doi:10.3390/mps2030077)
Supplement: Supplementary file 1 [file mps-02-00077-s001.pdf]

Supplementary material

Figure S1. Standard-care brochure Bangladesh.

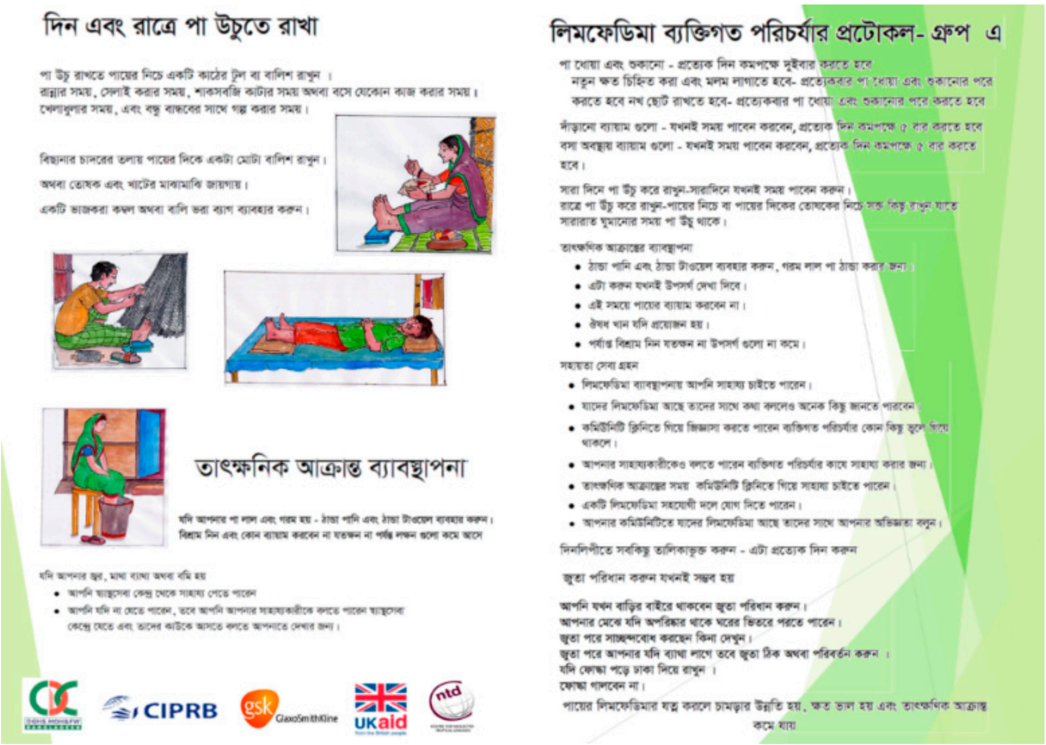

Front and back page view

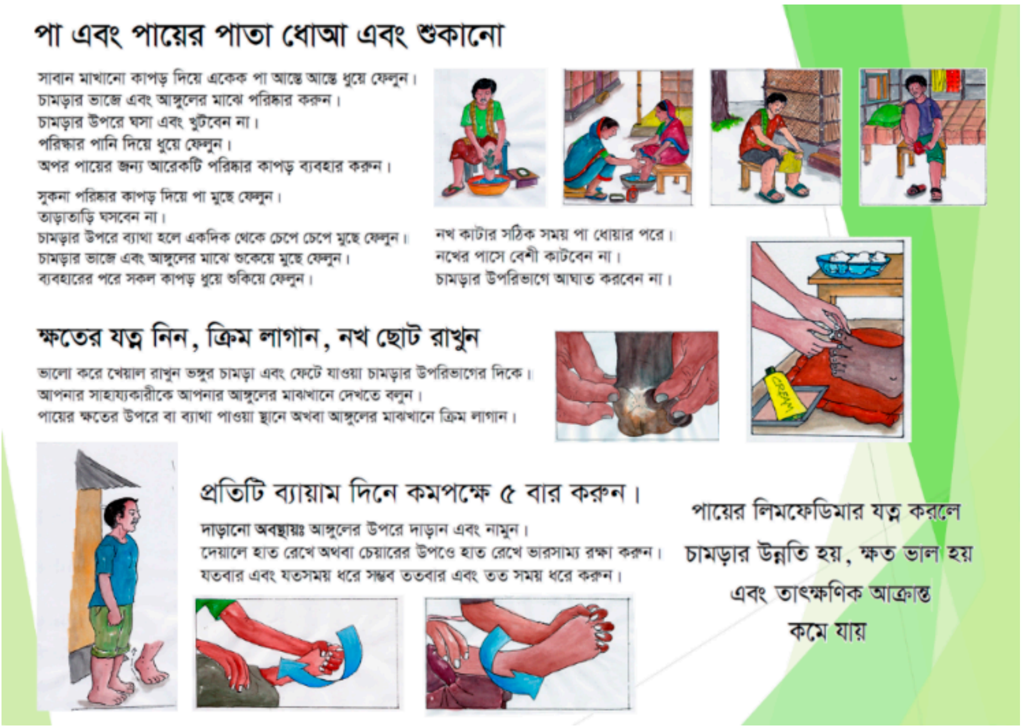

Inner page view

Figure S2. Enhanced-care brochure Ethiopia.

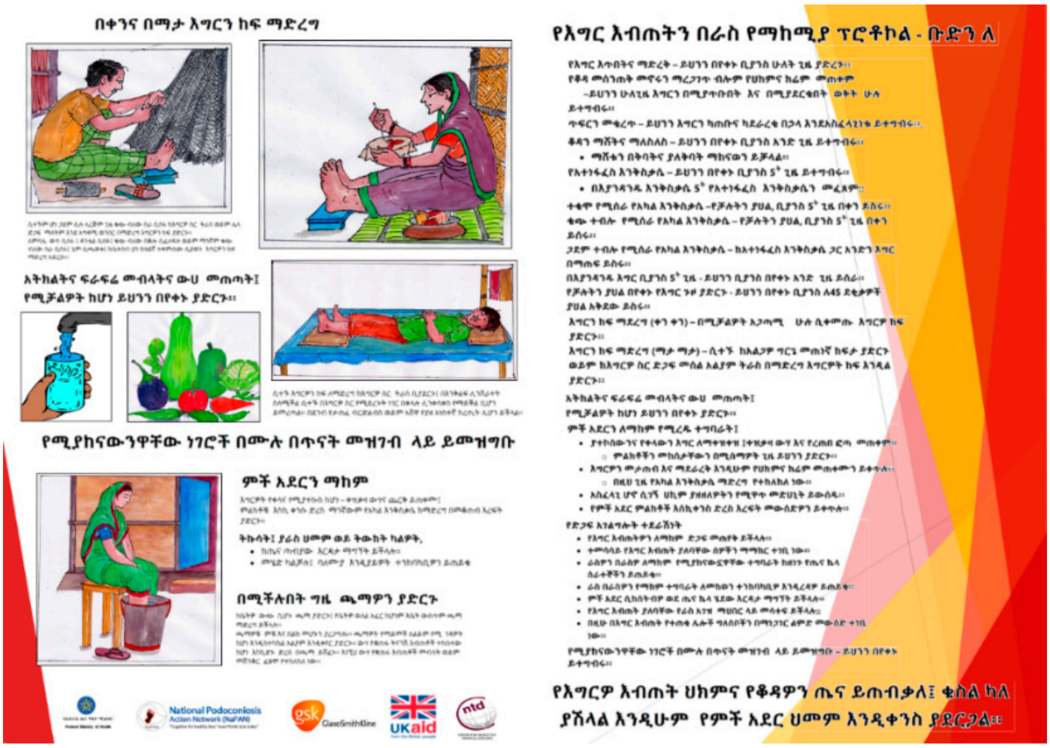

Front and back page view

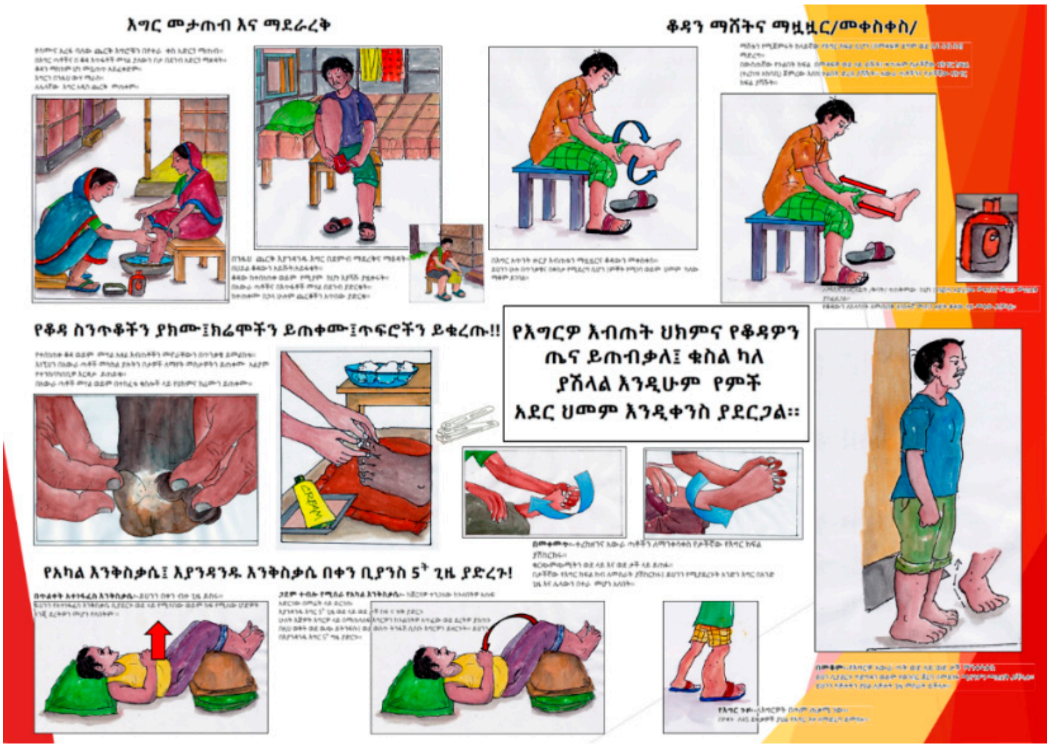

Inner page view
